# The complete chloroplast genome sequence of *Gynostemma yixingense* and comparative analysis with congeneric species

**DOI:** 10.1590/1678-4685-GMB-2020-0092

**Published:** 2020-09-25

**Authors:** Long Wang, Gengyu Lu, Hao Liu, Lijin Huang, Weimin Jiang, Ping Li, Xu Lu

**Affiliations:** 1 China Pharmaceutical University, School of Traditional Chinese Medicine, State Key Laboratory of Natural Medicines, Nanjing, China.; 2 Hunan Academy of Chinese Medicine, Institute of Chinese Materia Medica, Changsha, China.; 3 Hengyang Normal University, College of Life Sciences and Environment, Hengyang, Hunan, China.

**Keywords:** Chloroplast genome, Cucurbitaceae, Gynostemma, phylogeny, simple sequence repeats

## Abstract

*Gynostemma yixingense*, an important medicinal member of the Cucurbitaceae family, is an endemic herbaceous species distributed in East China. It is morphologically similar to the plants in the same genus, which resulted in some confusion in identification and application. Meanwhile, there are still some controversies in taxonomy. Herein, the complete chloroplast genome sequence of *G. yixingense* was obtained by Illumina paired-end sequencing technology and compared to other chloroplast genome sequences of congeneric species. The complete chloroplast genome of *G. yixingense* is 157,910 bp in length with 36.94% GC content and contains a large single-copy (LSC) region of 86,791 bp, a small single-copy (SSC) region of 18,635 bp and a pair of inverted repeat (IR) regions of 26,242 bp. The whole genome contains 133 unique genes, including 87 protein-coding genes, 37 tRNA genes, eight rRNA genes and one pseudogene. In addition, 74 simple sequence repeats (SSRs) were identified, most of which were A/T rich. The phylogenetic analysis indicated that *G. yixingense* had the closest relationship to *G. laxiflorum*. The result of this study provided an important theoretical basis for chloroplast genome and phylogenetic analysis of *G. yixingense*.

*Gynostemma yixingense,* a member of the *Gynostemma* genus belonging to the family Cucurbitaceae, is an endemic species in East China. It occurs in forests or thickets with an altitude of below 100 m, mainly produced in Anhui, southern Jiangsu and Zhejiang Province, China (Chen *et al.*, 2011). In 1990, *G. yixingense* var. *trichocarpum* was identified as a variant of *G. yixingense* (Ding, 1990). Chen (1995) classified the *Gynostemma* into two subgenera and two groups according to fruit type and style number. The berry type is of the *Gynostemma* subgenus, and the capsule type of the *Trirostellum* subgenus. The *Trirostellum* subgenus is further divided into two groups according to style number. The styles (4-) 5 is the *Pentastylos* group, and the styles 3 is the *Trirostellum* group. The original *G. yixingense* and *G. yixingense* var. *trichocarpum* were combined to *G. yixingense* in Flora of China (FOC). However, Jeffrey believed that *G. laxiflorum* and *G. yixingense* should be the same species in FOC. Today, the genus *Gynostemma* has 14 species (nine endemic) in China (Chen *et al.*, 2011; Zhang *et al.*, 2017).

For the existence of ginsenosides, *G. pentaphyllum,* the same genus of *G. yixingense* is known as ‘*Panax ginseng* of Southern China’ (Qin *et al.*, 2015). It is an important medicinal plant with a variety of therapeutic effects that include enhancing immunity, lowering cholesterol, regulating blood pressure, anti-inflammatory and anticancer (Wang *et al.*, 2017). *G. yixingense* contains similar chemical components to *G. pentaphyllum,* even containing higher ginsenosides (Zhang *et al.*, 2015). In addition, *G. yixingense* has a sweeter taste that makes it appreciated by consumers (Xiang *et al.*, 2010). *G. yixingense* is morphologically similar to *G. pentaphyllum*, which are often confused in identification and application.

Chloroplasts have relatively independent genetic material, the chloroplast genome. Compared with nuclear genes, the chloroplast genome is often more conserved, which is great significance in plant phylogeny and species identification (Zhou *et al.*, 2017; Meng *et al.*, 2018). Within *Gynostemma*, the complete chloroplast genomes of several plant species have been published (Zhang *et al.*, 2017, 2018; Shi *et al.*, 2019), nevertheless, no chloroplast genome of *G. yixingense* has been reported until now. Therefore, in order to provide a reference for systematic evolution and rational use of *G. yixingense*, the complete chloroplast genome of *G. yixingense* was sequenced and analyzed in this study. Phylogenetic analyses were performed with other nine plants in the same genus.

Fresh leaves of *G. yixingense* were collected from Tongwu village, Hangzhou City, Zhejiang Province, China, in bushes, 30°12’10’’ N, 120°03’04’’ E, elevation 237 m. Voucher specimens were deposited in the Center of Herbarium, China Pharmaceutical University, Nanjing, China, under accession number WL20191005. Whole genomic DNA was extracted from fresh leaves using Rapid Plant Genomic DNA Isolation Kit, Sangon Biotech (Shanghai). Then, the quality and integrity of DNA were checked using BioPhotometer Plus (Nucleic acid and protein detector, Eppendorf, Germany) and 1% agarose gels. High quality of DNA was used to construct the library. These raw reads were deposited in NCBI Sequence Read Archive (SRA) with the accession number PRJNA598798. Sequencing was performed on Illumina Xten platform (GENEWIZ Suzhou, China). Using the *G. pentaphyllum* chloroplast genome as reference (NCBI accession No. KX852298), clean reads of the matched reference genes were extracted. The obtained reads were assembled with NOVOPlasty v2.7.2 (Dierckxsen *et al.*, 2016), and the assembled genome was annotated and analyzed using the GeSeq tool (Tillich *et al.*, 2017). The chloroplast genome data was compared to the NCBI database by BLAST, searching for protein coding genes, rRNA genes and tRNA genes, while the tRNA genes were further confirmed using tRNAscan-SE v2.0 program (Lowe and Chan, 2016). The finally annotated chloroplast genome was deposited in GenBank with the accession number MT028489. The chloroplast genome map was drawn using OGDRAW v1.2 based on the annotated results (Lohse *et al.*, 2007).

After quality filtering, a total number of 65,730,414 clean reads (>Q20) were obtained, and the whole chloroplast genome was assembled using these clean reads. The length of the chloroplast genome of *G. yixingense* was 157,910 bp, which had the cyclic tetrad structure of chloroplast genomes typical in angiosperms, including large and small single copy regions (LSC and SSC) of 86,791 bp and 18,635 bp, respectively, and a pair of inverted repeats (IRa and IRb) of 26,242 bp. The overall GC content was 36.94%, and the GC content of the LSC, SSC and IR regions were 34.76%, 30.61% and 42.79%, respectively. In addition, a total of 133 genes were annotated, including 87 protein-coding genes, eight ribosomal RNA genes, 37 tRNA genes and one pseudogene (*infA*). Among them, 19 genes were duplicated in the IR regions, which contain eight protein-coding genes, seven tRNA genes and four rRNA genes (Figure 1, Table 1). The *rps12* gene had a trans-spliced structure. The 5’ and 3’ ends of *rps12* were located in the LSC region and IR region, respectively, which were divided into two independent transcription units. Furthermore, 15 genes possessed introns. Thirteen genes contained one intron, and two genes (*ycf3* and *clpP*) contained two introns. These introns ranged in length from 535 bp to 2,489 bp, of which *TrnK-UUU* gene had the longest intron, 2,489 bp (Table S1).

**Figure 1:**
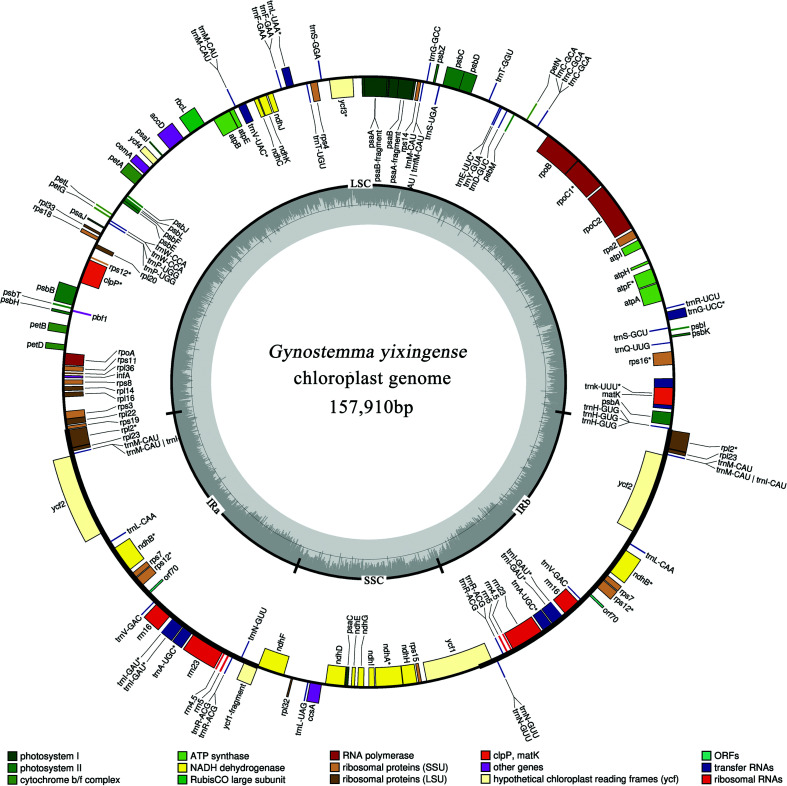
Chloroplast genome map of *Gynostemma yixingense*. Genes drawn inside the circle are transcribed clockwise, genes outside are transcribed counterclockwise. Genes are color coded by their function in the legend. The area in darker gray and lighter gray in the inner circle indicates GC content and AT content, respectively.

**Table 1:** Gene content in the chloroplast genome of of *Gynostemma yixingense.*

Category of genes	Group of genes	Name of genes
Photosynthesis	ATP synthase gene	atpA, atpB, atpE, atpF^*^, atpH, atpI
	NADH dehydrogenase	ndhA^*^, ndhB^*^(x2)*, ndhC, ndhD, ndhE, ndhF, ndhG, ndhH, ndhI, ndhJ, ndhK*
	Cytochrome b/f complex	*petA, petB, petD, petG, petN, petL*
	Photosystem I	*psaA, psaB, psaC, psaI, psaJ ycf4, ycf3* ^^*^^*^^
	Photosystem II	*psbH, psbN, psbT, psbE, psbZ, psbK, psbC, psbA, psbJ, psbL, psbI, psbM, psbF, psbB, psbD*
	Large chain of rubisco	*rbcL*
Self-replication	Large subunit of ribosome	rpl2^*^(x2)*, rpl14, rpl16, rpl20, rpl22, rpl23*(x2) *, rpl32, rpl33, rpl36*
	RNA polymerase subunits	rpoA, rpoB, rpoC1^*^, rpoC2
	Small subunit of ribosome	*rps2, rps3, rps4, rps7*(x2), rps8, rps11, rps12^*^(x2) , rps14, rps15, rps16^*^, rps18, rps19
	rRNA genes	*rrn23*(x2)*, rrn4.5*(x2)*, rrn5*(x2)*, rrn16*(x2)
	tRNA genes	*trnR-ACG*(x2)*, trnN-GUU*(x2)*, trnV-GAC*(x2)*, trnL-CAA*(x2)*, trnE-UUC, trnY-GUA, trnD-GUC, trnR-UCU, trnI-CAU*(x2), trnP-UGG, trnM-CAU, trnF-GAA, trnH-GUG, trnC-GCA, trnS-UGA, trnV-UAC^*^, trnT-GGU, trnQ-UUG, trnG-GCC, trnS-GGA, trnG-UCC^*^, trnI-GAU^*^(x2), trnA-UGC^*^(x2), trnk-UUU^*^, trnfM-CAU, trnS-GCU, trnT-UGU, trnL-UAA^*^, trnL-UAG, trnW-CCA
Other genes	Acetyl-CoA carboxylase	*accD*
	Cytochrome c biogenesis	*ccsA*
	Membrane protein	*cemA*
	ATP-dependent protease	*clpP* ^^*^^*^^
	Maturase	*matK*
	Translational initiation factor	^*ψ*^ *infA*
Unknown function	Conserved open reading frames	*ycf2(*x2*), ycf1(*x2*)*
	hypothetical chloroplast protein	*orf70*(x2)

* Indicates the genes containing one intron

** Indicates the genes containing two introns, (x2) Indicates genes duplicated in the IR regions.

ψ Indicates the pseudogene.

The length of angiosperm chloroplast genomes is variable primarily due to expansion and contraction of IR region (Zhang *et al.*, 2014). Hence, the IR/SC boundary regions of the ten *Gynostemma* chloroplast genomes were compared in this study (Figure 2). The results showed, except some boundary differences, all the ten *Gynostemma* chloroplast genomes exhibited striking similarities on the IR borders. For example, *rps19* across the IRb/LSC boundary in *G. pentagyum, G. compressum, G. laxiflorum and G. caulopterum* in IRb/LSC region, while the other six species were situated in the LSC region. The SSC/IRb boundary of all plants were located in the *ycf1* gene, resulting in the production of the *ycf1* pseudogene in the IRa region. In the IRa/SSC region, *ycf1* gene across all the IRa/SSC boundaries, but the fragment length of *ycf1* genes in SSC region were different.

**Figure 2:**
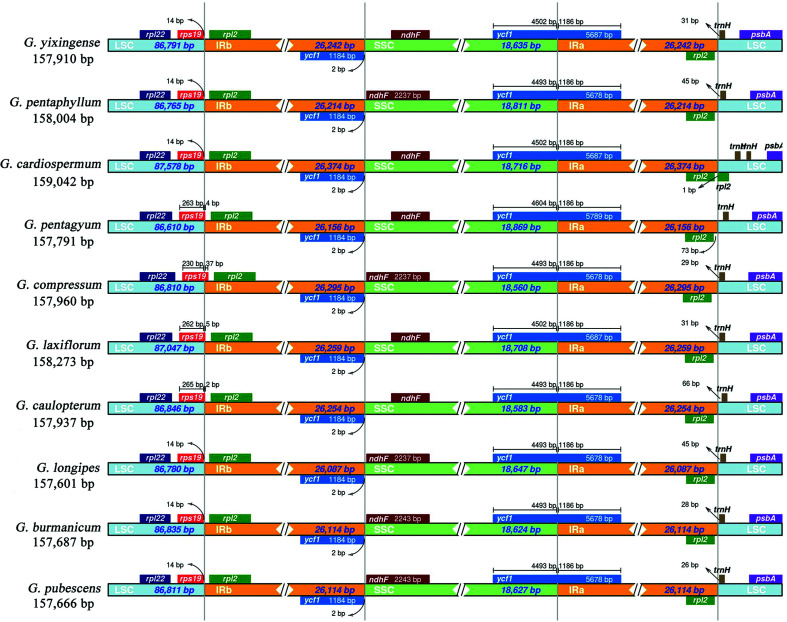
Comparison of the LSC, IR and SSC junction positions among ten Gynostemma chloroplast genomes. Colored boxes for genes represent the gene position.

Simple sequence repeats (SSRs), also known as microsatellites, are short repeat sequences with length of 1-6 bp, which are widely used in phylogenetic analysis and population genetics (Cavalier-Smith, 2002). In this study, the microsatellite identification tool MISA (https://webblast.ipk-gatersleben.de/misa/) was used to detect SSRs (parameter setting: the minimum repeat number of 10, 5, 4, 3, 3 and 3 for mono-, di-, tri-, tetra-, penta- and hexanucleotide repeats, respectively). The maximum length of sequence between two SSRs to register as compound SSR was 100 bp. A total of 74 SSRs were identified in the chloroplast genome of *G. yixingense*, including 46 mononucleotide repeats (62.2%), 16 dinucleotide repeats (21.62%), three trinucleotide repeats (4.05%), and nine tetranucleotide repeats (12.16%). There were 67 SSRs made up of A or T (90.54%), which indicates that the composition of SSRs tends to use A/T (Table S2).

To construct a phylogenetic tree, 13 chloroplast genomes from Cucurbitaceae were employed, including *G. yixingense*, nine other *Gynostemma* species and three outgroups (Table S3). The criterion for selection of outgroups was that they should be medicinal plants in a different genus of Cucurbitaceae and plants with relatively similar morphology. The chloroplast genomes of the selected species were downloaded from NCBI. Phylogenetic inference was performed using 77 common protein-coding genes (Table S4). MAFFT (Katoh and Standley, 2013) was employed to sequence alignment, and BioEdit v7.0.9.0 (Hall, 1999) was also used to examine and manually adjust the sequence alignment result. Phylogenetic trees were constructed using Maximum Parsimony (MP) and Bayesian Inference (BI) analysis. MP analysis was performed in PAUP* v4.0 beta 10 (Swofford, 2002), and BI was performed in MrBayes 3.2.6 (Ronquist *et al.*, 2012). For the MP analysis, the bootstrap probability was determined with 1000 replicates. For BI analysis, the best-fit model (GTR+I+G) in the analysis was selected by Akaike information criterion (AIC) in MrModeltest v2.3 (Nylander, 2004). Four Markov Chains Monte Carlo (MCMC) samples were run for 1 × 10^6^ generations. The convergence of MCMC runs was additionally confirmed by two independent runs, and trees were sampled every 100 generations. The burn-in was set to discard 25% of the trees to produce consensus tree of all remaining trees.

The results of molecular analysis based on MP and BI methods showed the same topology (Figure 3). All species of *Gynostemma* were clustered into one monophyletic clade with a high bootstrap support value, which were divided into two subclades. The first subclade consisted of the Subgen. *Gynostemma* except G. *pentagynum*, while the other subclade consisted of the Subgen. *Trirostellum*. It is basically consistent with the morphological classification by Chen (1995). G. *pentagynum* has styles (4-) 5, which differs from other species of *Gynostemma. G. yixingens*e had the closest phylogenetic relationship to *G. laxiflorum*, which formed a clade and had a close phylogenetic relationship to the subclade of *G. cardiospermum*. We had already done a number of field resource surveys, and did not discover *G. laxiflorum.* *G. laxiflorum* and *G. yixingense* could be the same species in FOC. Combining the geographical distribution, morphological characteristics and molecular phylogeny, we consider that the taxonomy of *G. laxiflorum* and *G. yixingense* still needs further study.

**Figure 3:**
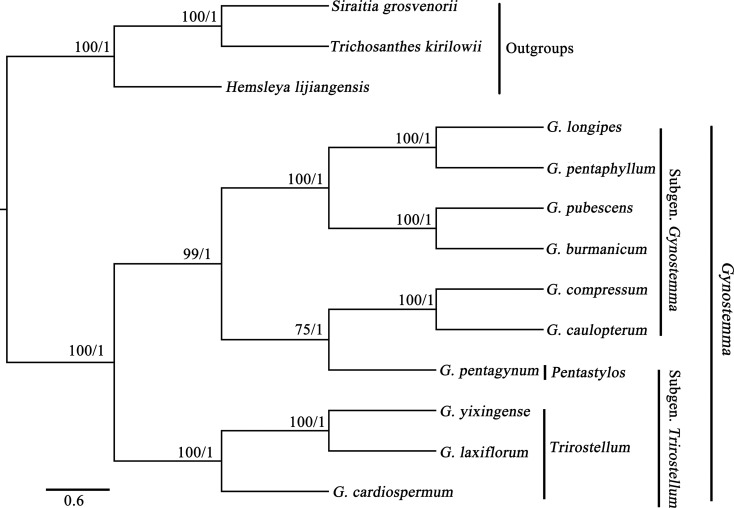
The Bayesian Phylogenetic tree based on ten *Gynostemma* species. Numbers above the branches represent MP bootstrap (BS) and Bayesian posterior probabilities (PP) values.

Overall, the complete chloroplast genome sequence of *G. yixingense* was reported and analyzed. Comparing with chloroplast genomes of other *Gynostemma*, the chloroplast genome of *G. yixingense* was conserved and very similar to other *Gynostemma* species. Phylogenetic analysis indicated that *G. yixingense* has the closest phylogenetic relationship to *G. laxiflorum*. The repeat sequences could be usted for developing genetic markers. The data in this study provided a useful tool for molecular identification and evolutionary studies in *Gynostemma*.

## Supplementary Material

The following online material is available for this article:

Table S1 Information of gene introns in the chloroplast genome of *Gynostemma yixingense.*

Table S2 Types and numbers of SSR identified in the chloroplast genome of *Gynostemma yixingense.*

Table S3 Chloroplast genome sequences used for phylogenetic tree construction.

Table S4 List of 77 chloroplast protein coding genes used of in the phylogenetic analysis.

## References

[B1] Chen SK, Lu AM, Jeffrey C (2011). Flora of China.

[B2] Chen SK (1995). A classificatory system and geographical distribution of the genus *Gynostemma* BL. (Cucurbitaceae). Acta Phytotaxonomica Sinica.

[B3] Cavalier-Smith T (2002). Chloroplast evolution: secondary symbiogenesis and multiple losses. Curr Biol.

[B4] Ding JG (1990). A new variety of *Gynostemma yixingese*. Bulletin of botanical research.

[B5] Dierckxsens N, Mardulyn P, Smits G (2016). NOVOPlasty: De novo assembly of organelle genomes from whole genome data. Nucleic Acids Res.

[B6] Hall TA (1999). BioEdit: A user-friendly biological sequence alignment editor and analysis program for Windows 95/98/NT. Nucleic Acids Symp Ser.

[B7] Katoh K, Standley DM (2013). MAFFT Multiple Sequence Alignment Software Version 7: Improvements in performance and usability. Mol Biol Evol.

[B8] Lowe TM, Chan PP (2016). tRNAscan-SE On-line: integrating search and context for analysis of transfer RNA genes. Nucleic Acids Res.

[B9] Lohse M, Drechsel O, Bock R (2007). OrganellarGenomeDRAW (OGDRAW): a tool for the easy generation of high-quality custom graphical maps of plastid and mitochondrial genomes. Curr Genet.

[B10] Meng J, Li XP, Li HT, Yang JB, Wang H, He J (2018). Comparative analysis of the complete chloroplast genomes of four aconitum medicinal species. Molecules.

[B11] Nylander JAA (2004). *MRMODELTEST version 2.1.* Computer program distributed by the author.

[B12] Qin SS, Li HT, Wang ZY, Cui ZH, Yu LY (2015). Analysis phylogenetic relationship of *Gynostemma* (Cucurbitaceae). China J Chin Mater Med.

[B13] Ronquist F, Teslenko M, Van DMP, Ayres DL, Darling A, Sebastian H, Larget B, Liu L, Suchard MA, Huelsrnbeck JP (2012). Mrbayes3.2: Efficient bayesian phylogenetic inference and model choice across a large model space. Syst Biol.

[B14] Shi YC, Zou R, Liu BB (2019). Complete chloroplast genome sequence of *Gynostemma pentaphyllum* (Cucurbitaceae), a perennial medicinal herb. Mitochondrial DNA B.

[B15] Swofford DL (2002). PAUP*: Phylogenetic Analysis using Parsimony (* and Other Methods).

[B16] Tillich M, Lehwark P, Pellizzer T, Ulbricht-Jones ES, Fischer A, Bock R, Greiner S (2017). GeSeq-versatile and accurate annotation of organelle genomes. Nucleic Acids Res.

[B17] Wang J, Yang JL, Zhou PP, Meng XH, Shi YP (2017). Further New Gypenosides from Jiaogulan (*Gynostemma pentaphyllum*). J Agric Food Chem.

[B18] Xiang WJ, Guo CY, Ma L, Hu LH (2010). Dammarane-type glycosides and long chain sesquiterpene glycosides from *Gynostemma yixingense*. Fitoterapia.

[B19] Zhang T, Liu WP, Li H, Xiao YP (2015). Ecological distribution and utilization of *Gynostemma pentaphyllum* germplasm resources in China. Shaanxi J Agric Sci.

[B20] Zhang X, Li HM, Zhou T, Yang YC, Zhao GF (2018). Characterization of the complete chloroplast genome sequence of *Gynostemma compressum* (Cucurbitaceae), an endemic plant in China. Conserv Genet Resour.

[B21] Zhang X, Zhou T, Kanwal N, Zhao YM, Bai GQ, Zhao GF (2017). Completion of Eight *Gynostemma* BL. (Cucurbitaceae) Chloroplast Genomes: Characterization, Comparative Analysis, and Phylogenetic Relationships. Front Plant Sci.

[B22] Zhang Y, Li L, Liang T, Liu Q (2014). Complete chloroplast genome sequences of *Praxelis* (*Eupatorium catarium Veldkamp*), an important invasive species. Gene.

[B23] Zhou JG, Chen XL, Cui YX, Sun W, Li YH, Wang Y, Song JY, Yao H (2017). Molecular structure and phylogenetic analyses of genomes of two aristolochia medicinal species. Int J Mol Sci.

