# Supplementary material for: The complete chloroplast genome sequence of Gynostemma yixingense and comparative analysis with congeneric species
Source: Genet Mol Biol. 2020 Sep 25;43(4):e20200092. doi: 10.1590/1678-4685-GMB-2020-0092 (PMC7521087; doi:10.1590/1678-4685-GMB-2020-0092)
Supplement: Supplementary file 1 [file 1415-4757-GMB-43-4-e20200092-suppl1.pdf]

Genetics and Molecular Biology
https://doi.org/10.1590/1678-4685-GMB-2020-0092

Supplementary Material to "The complete chloroplast genome sequence of Gynostemma yixingense and comparative analysis with congeneric species".

Table S1 - Information of gene introns in the chloroplast genome of *Gynostemma yixingense.*

Genes	Location	Length/bp				
		Exon I	Intron I	Exon II	Intron II	Exon III
atpF	LSC	411	688	144		
ndhB	IR	756	686	777		
ndhA	SSC	541	1176	551		
rpl2	IR	435	682	393		
rpoC1	LSC	1611	725	432		
*rps12**	LSC-IR	114	—	231	540	27
rps16	LSC	209	850	44		
trnV-UAC	LSC	37	601	39		
trnG-UCC	LSC	20	659	51		
trnI-GAU	IR	42	952	35		
trnA-UGC	IR	37	802	36		
trnk-UUU	LSC	37	2489	35		
trnL-UAA	LSC	38	535	48		
ycf3	LSC	155	746	226	716	126
clpP	LSC	228	622	291	835	69

* trans-spliced gene.
