# Supplementary material for: The complete chloroplast genome sequence of Gynostemma yixingense and comparative analysis with congeneric species
Source: Genet Mol Biol. 2020 Sep 25;43(4):e20200092. doi: 10.1590/1678-4685-GMB-2020-0092 (PMC7521087; doi:10.1590/1678-4685-GMB-2020-0092)
Supplement: Supplementary file 2 [file 1415-4757-GMB-43-4-e20200092-suppl2.pdf]

Genetics and Molecular Biology
https://doi.org/10.1590/1678-4685-GMB-2020-0092

Supplementary Material to "The complete chloroplast genome sequence of Gynostemma yixingense and comparative analysis with congeneric species".

Table S2 - Types and numbers of SSR identified in the chloroplast genome of *Gynostemma yixingense.*

Repeat motif	Type of repeats	Number of repeats	Ratio
mononucleotide	A	20	62.2%
	G	1	
	T	25	
dinucleotide	AG	1	21.62%
	AT	3	
	CT	1	
	TA	10	
	TC	1	
trinucleotide	AAT	1	4.05%
	GAT	1	
	TAT	1	
tetranucleotide	AAAT	1	12.16%
	AAGT	1	
	AATA	1	
	ATTT	1	
	CAAA	1	
	CCCA	1	
	TAAA	3	
