# Supplementary material for: The complete chloroplast genome sequence of Gynostemma yixingense and comparative analysis with congeneric species
Source: Genet Mol Biol. 2020 Sep 25;43(4):e20200092. doi: 10.1590/1678-4685-GMB-2020-0092 (PMC7521087; doi:10.1590/1678-4685-GMB-2020-0092)
Supplement: Supplementary file 3 [file 1415-4757-GMB-43-4-e20200092-suppl3.pdf]

Genetics and Molecular Biology
https://doi.org/10.1590/1678-4685-GMB-2020-0092

Supplementary Material to "The complete chloroplast genome sequence of Gynostemma yixingense and comparative analysis with congeneric species".

Table S3- Chloroplast genome sequences used for phylogenetic tree construction

Species name	GenBank ID	Species name		GenBank ID
Gynostemma yixingense	MT028489	*G. cardiospermum*		KX852299
G. longipes	MF152730	*G. caulopterum*		MF136487
G. burmanicum	MF152731	*G. compressum*		KY817143
G. laxiflorum	MF136486			
G. pentagynum	KY670737	Outgroups	*Trichosanthes kirilowii*	MK036046
G. pentaphyllum	KX852298		*Siraitia grosvenorii*	MK818498
G. pubescens	MF152732		*Hemsleya lijiangensis*	MG733988
