# Supplementary material for: The complete chloroplast genome sequence of Gynostemma yixingense and comparative analysis with congeneric species
Source: Genet Mol Biol. 2020 Sep 25;43(4):e20200092. doi: 10.1590/1678-4685-GMB-2020-0092 (PMC7521087; doi:10.1590/1678-4685-GMB-2020-0092)
Supplement: Supplementary file 4 [file 1415-4757-GMB-43-4-e20200092-suppl4.pdf]

Genetics and Molecular Biology
https://doi.org/10.1590/1678-4685-GMB-2020-0092

Supplementary Material to "The complete chloroplast genome sequence of Gynostemma yixingense and comparative analysis with congeneric species".

Table S4 - List of 77 chloroplast protein coding genes used of in the phylogenetic analysis.

Gene	Name	Gene	Name
accD	acetyl-CoA carboxylase beta subunit	psbH	photosystem II phosphoprotein
atpA	ATP synthase CF1 alpha chain	psbI	photosystem II protein I
atpB	ATP synthase CF1 beta subunit	psbJ	photosystem II protein J
atpE	ATP synthase CF1 epsilon subunit	psbK	photosystem II protein K
atpF	ATP synthase CF0 B subunit	psbL	photosystem II protein L
atpH	ATP synthase CF0 C chain	psbM	photosystem II protein M
atpI	ATP synthase CF0 A chain	psbN	photosystem II protein N
ccsA	cytochrome c biogenesis protein	psbT	photosystem II protein T
cemA	envelope membrane protein	psbZ	photosystem II protein Z
clpP	ATP-dependent Clp protease proteolytic subunit	rbcL	ribulose-1,5-bisphosphate carboxylase/oxygenase large subunit
matK	maturase K	rpl2	ribosomal protein L2
ndhA	NADH dehydrogenase subunit A	rpl14	ribosomal protein L14
ndhB	NADH dehydrogenase subunit B	rpl16	ribosomal protein L16
ndhC	NADH dehydrogenase subunit C	rpl20	ribosomal protein L20
ndhD	NADH dehydrogenase subunit D	rpl22	ribosomal protein L22
ndhE	NADH dehydrogenase subunit E	rpl23	ribosomal protein L23
ndhF	NADH dehydrogenase subunit F	rpl32	ribosomal protein L32
ndhG	NADH dehydrogenase subunit G	rpl33	ribosomal protein L33
nhdH	NADH dehydrogenase subunit H	rpl36	ribosomal protein L36
ndhI	NADH dehydrogenase subunit I	rpoA	RNA polymerase alpha subunit
ndhJ	NADH dehydrogenase subunit J	rpoB	RNA polymerase beta subunit
ndhK	NADH dehydrogenase subunit K	rpoC1	RNA polymerase beta subunit-1
petA	component of cytochrome b6/f complex	rpoC2	RNA polymerase beta subunit-2
petB	cytochrome b6	rps2	ribosomal protein S2
petD	cytochrome b6/f complex subunit IV	rps3	ribosomal protein S3
petG	cytochrome b6/f complex subunit V	rps4	ribosomal protein S4
petL	cytochrome b/f complex 3.5 kDa subunit	rps7	ribosomal protein S7
petN	cytochrome b6/f complex subunit VIII	rps8	ribosomal protein S8
psaA	photosystem I P700 apoprotein A1	rps11	ribosomal protein S11
psaB	photosystem I P700 apoprotein A2	rps12	ribosomal protein S12
psaC	photosystem I subunit VII	rps14	ribosomal protein S14

Gene	Name	Gene	Name
psaI	photosystem I subunit VIII	rps15	ribosomal protein S15
psaJ	photosystem I subunit IX	rps16	ribosomal protein S16
psbA	photosystem II protein D1	rps18	ribosomal protein S18
psbB	PSII 47 kDa protein	rps19	ribosomal protein S19
psbC	photosystem II 43 kDa protein	ycf1	photosystem I assembly protein Ycf1
psbD	photosystem II protein D2	ycf2	hypothetical chloroplast RF2
psbE	photosystem II protein V	ycf3	photosystem I assembly protein Ycf3
psbF	photosystem II cytochrome b559 beta subunit		
